# Multi-Output Prediction and Optimization of CO_2_ Laser Cutting Quality in FFF-Printed ASA Thermoplastics Using Machine Learning Approaches

**DOI:** 10.3390/polym17141910

**Published:** 2025-07-10

**Authors:** Oguzhan Der

**Affiliations:** Marine Engineering Department, Bandirma Onyedi Eylul University, 10200 Balikesir, Türkiye; oder@bandirma.edu.tr

**Keywords:** laser cutting, additive manufacturing, ASA thermoplastic, surface quality, kerf width, heat-affected zone, machine learning

## Abstract

This research article examines the CO_2_ laser cutting performance of Fused Filament Fabricated Acrylonitrile Styrene Acrylate (ASA) thermoplastics by analyzing the influence of plate thickness, laser power, and cutting speed on four quality characteristics: surface roughness (Ra), top kerf width (Top KW), bottom kerf width (Bottom KW), and bottom heat-affected zone (Bottom HAZ). Forty-five experiments were conducted using five thickness levels, three power levels, and three cutting speeds. To model and predict these outputs, seven machine learning approaches were employed: Autoencoder, Autoencoder–Gated Recurrent Unit, Autoencoder–Long Short-Term Memory, Random Forest, Extreme Gradient Boosting (XGBoost), Support Vector Regression, and Linear Regression. Among them, XGBoost yielded the highest accuracy across all performance metrics. Analysis of Variance results revealed that Ra is mainly affected by plate thickness, Bottom KW by cutting speed, and Bottom HAZ by power, while Top KW is influenced by all three parameters. The study proposes an effective prediction framework using multi-output modeling and hybrid deep learning, offering a data-driven foundation for process optimization. The findings are expected to support intelligent manufacturing systems for real-time quality prediction and adaptive laser post-processing of engineering-grade thermoplastics such as ASA. This integrative approach also enables a deeper understanding of nonlinear dependencies in laser–material interactions.

## 1. Introduction

Additive manufacturing (AM), commonly known as three-dimensional (3D) printing, has gained significant attention in the past few decades due to its potential for fabricating complex geometries, reducing material waste, and enabling rapid prototyping [1]. AM technologies have shaken the original production paradigms within virtually every industry, including aerospace, automobile, biomedical, and consumer electronics [2]. Of the many processes that constitute additive manufacturing, Fused Filament Fabrication (FFF) scores a little higher owing to cost-effectiveness, availability of materials, and ease of use [3]. It entails processing thermoplastic filaments layer-wise to fabricate three-dimensional objects directly from digital model data. This technology has gained tremendous popularity in industrial and research environments for making mainly functional prototypes and custom components. Against this backdrop, the demand for high-performance thermoplastics in FFF has surged, leading to the adoption of engineering-grade polymers such as Acrylonitrile Styrene Acrylate (ASA) [4].

ASA is an outdoor-use thermoplastic with good weathering and ultraviolet (UV) stability; it also exhibits improved mechanical strength and temperature stability over other thermoplastics such as polyvinyl chloride (PVC), polycarbonate (PC), polypropylene (PP), and polyethylene (PE) [5]. Its resistance to the outdoor environment makes it perfect for outdoor applications, automotive parts, and technical housing [6]. Using ASA with the FFF process allows for the manufacture of parts that need mechanical strength and good finishes. On the other hand, FFF processes have some apparent drawbacks, including limited dimensional accuracy, interlayer adhesion problems, and surface roughness-related issues caused by the layer-wise deposition process [7]. Hence, post-processing measures are required to improve the printed components’ functional and aesthetic attributes. Among many methods, laser cutting is gaining prominence as a precise and reasonably fast process for correcting geometry, improving surface finishes, and customizing components [8].

The laser cutting method is an advanced manufacturing technique that is widely used due to its high accuracy, excellent flexibility, and capability to process a variety of materials, including metals, polymers, and composites [9]. CO_2_ laser cutting is more often used for thermoplastics because of its conformity with polymer absorption spectra and ability to provide clean cuts without any mechanical stress. The process has the benefit of reduced tool wear, narrow heat-affected zones (HAZs), and possibilities for automation [10]. The CO_2_ laser cutting process is a very important setup in industries where polymer-based components are involved, such as the production of labels, packaging, automotive interiors, and medical devices [11].

Numerous studies have recently been published on CO_2_ laser machining for FFF-based thermoplastics such as polylactic acid (PLA), acrylonitrile butadiene styrene (ABS), and polyethylene terephthalate glycol (PETG), with surface roughness (Ra), kerf width (KW), kerf angle, and HAZ as key parameters to be optimized. For example, Kechagias et al. [12] investigated the laser processing of PLA plates produced via FFF and found that kerf angle and surface roughness were greatly influenced by laser power and cutting speed. Their investigation further showed that neural networks and genetic algorithms could be used in the modeling and optimization of laser cutting results. Contrary to this, while analyzing PETG from various angles, Kechagias et al. [13] tested experimental and machine learning methods, including feed-forward back-propagation neural networks, to predict surface roughness and kerf characteristics, confirming the possibility of intelligent prediction models for CO_2_ laser cutting of FFF materials. Another fine attempt by Sabri et al. [14] focused on sheets of PETG of different thicknesses and studied the effects of cutting speed, laser power, and sheet thickness on upper/lower kerf widths and the HAZ. Their study employed RSM and provided valuable insights into thermal–material interactions in polymer laser cutting. However, the study remained confined to PETG and did not examine other engineering-grade thermoplastics such as ASA. In addition, Kechagias et al. [15] highlighted the potential of hybrid modeling methods that consider neural networks and grey wolf optimization to improve the kerf geometry and surface roughness of FFF PLA parts. Most importantly for the present investigation, Kechagias et al. [7] made one of the few pioneering contributions to the CO_2_ laser cutting of 3D-printed ASA plates. Their study evaluated three ASA sheet thicknesses for varying focal distances and cutting speeds. The study set up a Box–Behnken experimental design to predict upper KW, lower KW, and Ra using regression models. The proposed low-power laser systems were considered suitable for ASA post-processing. However, with limited input parameters and performance metrics range considered during the study, the scope remained narrow. While FFF materials have been moderately studied relative to laser cutting, the literature remains limited for multi-parameter and multi-output analyses of 3D-printed ASA, which is triple-varied by power, speed, and thickness. This research bridges that gap by studying five thickness levels of ASA under varying laser strengthening conditions, analyzing multiple quality criteria (Ra, Top/Bottom KW, Bottom HAZ), and adopting the machine learning paradigm for predictive modeling and process optimization.

Therefore, this study intends to fill this research gap via a systematic study of the laser cutting behavior of 3D-printed ASA samples at five thicknesses, three powers, and three speeds. Departing from most of the previous work, in which only one or two quality indicators were studied, this paper studies Ra, Top KW, Bottom KW, and Bottom HAZ simultaneously to provide a multifaceted view of cut quality. Furthermore, the performance characteristics are subjected to predictive modeling via machine learning models, enabling an evaluation of the predictive accuracy and the identification of the most influential input factors. Thus, by combining extensive experiments and state-of-the-art modeling, this study enriches the literature with novel scientific findings and offers valuable insights to practitioners aiming to optimize laser processing of 3D-printed ASA parts.

## 2. Materials and Methods

### 2.1. ASA Sample Production

ASA filament, 1.75 mm in diameter and commercially sourced from Filameon Company (Kayseri, Türkiye), was used to fabricate the experimental specimens. ASA is an engineering thermoplastic resistant to weather with good mechanical strength and UV resistance. For outdoor applications, ASA is the filament of choice over a standard filament because it offers higher dimensional stability, heat resistance, and a better finish. Printing was conducted on a 3D printer using the FFF technique under optimal conditions to obtain flawless quality and layer adhesion.

**Figure 1:**
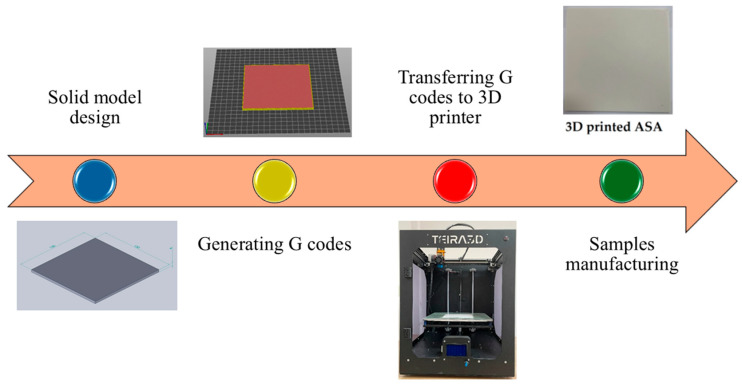
Flowchart of samples manufactured by AM.

The laser-cutting specimens, each with surface dimensions of 130 × 130 mm^2^ and thicknesses ranging from 2 mm to 4 mm in 0.5 mm increments, were designed using SolidWorks CAD software (version 2020). G-codes were then converted with PrusaSlicer 2.6.1 and were 3D-printed with a TEIRA3D printer (TEIRA3D, Kocaeli, Türkiye), which works on the principles of FFF technology. The various stages of sample production on the 3D printer are explained in detail in Figure 1.

Preliminary experiments were conducted to identify suitable 3D printing parameters for the successful production of ASA samples. The fixed production parameters used in 3D printing were fill density (100%), orientation angle (±45 degree), nozzle temperature (250 °C), table temperature (100 °C), fill pattern (line), number of wall lines (3), top layers (5), print speed (40 mm/s), bottom layers (4), and fan speed (100%).

### 2.2. Laser Cutting

Laser cutting of the 3D-printed ASA specimens was carried out using a LazerFix LF7010 CNC-controlled Laser Cutting Machine (Konya, Türkiye) equipped with a CO_2_ laser and a maximum power of 100 W. The distance between the laser nozzle and the workpiece was kept constant at 7 mm to ensure repeatability and reproducibility under all experimental runs. This distance was optimized considering the proper focusing of the beam and smooth ejection of molten material from the cutting area. Compressed air in the setup aided in removing molten ASA from the cutting zone. Simultaneously, it prevented contamination of the focusing optics by dust and debris produced during the cutting process. Plate thickness, cutting speed, and power were selected as the main cutting parameters, each varied at three different levels based on preliminary trials and literature that commonly address CO_2_ laser cutting of thermoplastics. Table 1 provides an overview of the control factors and levels used.

**Figure 2:**
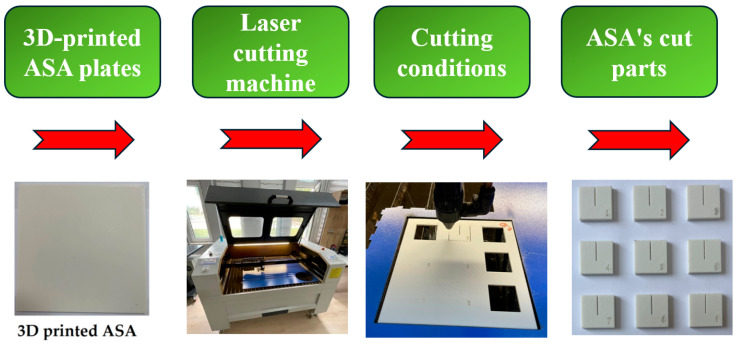
Flowchart of CO_2_ laser cutting.

During the cutting operation, the 3D-printed ASA plate was clamped onto the CNC table using blue PP material for stabilization. ASA plates measuring 130 mm × 130 mm were used in the laser cutting process. From these plates, nine square samples with dimensions of 25 mm × 25 mm were obtained through laser cutting. To enable measurements on the cut samples, a 15 mm long slit was created at the center of each sample. The flowchart of the laser cutting process for the 3D-printed ASA samples is shown in Figure 2. After the cutting operation, Ra and KW were measured for each of the samples to evaluate how different cutting parameters affected the cut quality. The measurements were later analyzed to select laser-cutting parameters that could minimize Ra while maintaining dimensional accuracy at levels as high as possible.

### 2.3. Measurement of Responses

**Figure 3:**
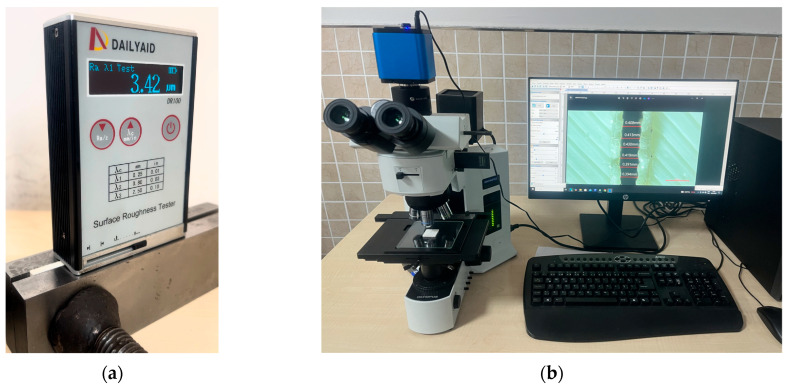
Measurement tools employed in the study: (**a**) roughness tester, (**b**) microscope.

To investigate the influence of laser cutting parameters, Ra, Top/Bottom KW, and Bottom HAZ were chosen as the response variables and measured accordingly. Samples measuring 25 × 25 mm were secured between two precision-ground metal blocks to ensure parallel alignment along a common axis. This allowed for proper Ra measurements. Nine Ra measurements in total were taken on platforms, three on each of the three cut edges, which were subsequently averaged. Ra values were measured by DAILYAID DR100 Surface Roughness Tester (Beijing Dailyaid Measuring & Control Ltd., Beijing, China). The ISO 4288 standard was used to analyze the samples, with a cut-off wavelength of 0.8 mm and an evaluation length of 4 mm [16]. The formation of a cross-section on the cut specimens was necessary for measuring Top/Bottom KW and Bottom HAZ. These were measured using an OLYMPUS brand and BX51 model light microscope (OLYMPUS OPTICAL CO., LTD., Tokyo, Japan). A total of four, six, and eight measurements were evaluated for Top/Bottom KW and Bottom HAZ, respectively, and their mean values were computed. Instruments used in the evaluation of quality characteristics are shown in Figure 3. Moreover, Analysis of Variance (ANOVA) was employed to determine whether any of the process parameters, such as plate thickness, laser power, and cutting speed, make a significant contribution to the quality measures. ANOVA at 95% confidence was carried out for the F-values and *p*-values to find the most essential factor for each output variable.

## 3. Machine Learning Models

The methodological framework of machine learning (ML) modeling involves predicting experimental data to evaluate the effects of different CO_2_ laser cutting parameters on the responses. Data was provided for 45 different parameter combinations, considering three independent and four dependent variables for each experimental setup. During the data preprocessing phase, all input and output variables were normalized using the StandardScaler function. Subsequently, the dataset was randomly divided into training and testing sets, with 80% allocated for training and the remaining 20% for testing, ensuring an unbiased evaluation [17]. For Recurrent Neural Network (RNN)-based models of Long Short-Term Memory (LSTM) and Gated Recurrent Unit (GRU), the data needed to be repackaged so that it would have a temporal nature (samples, steps, and features) for time-dependent analysis. The study considered seven different ML models comprising hybrid models based on deep learning (DL): Autoencoder (AE), Autoencoder–GRU (AE-GRU), and Autoencoder–LSTM (AE-LSTM). The AE model performs dimensionality reduction by compressing data while preserving key nonlinear patterns within the dataset [18]. The AE-GRU model combines the encoding capability of autoencoders with the sequence learning strength of GRUs, effectively capturing both temporal and marginal dependencies while addressing the vanishing gradient issue common in RNNs [19]. Similarly, the AE-LSTM model enhances the representation of long-term dependencies, delivering improved performance in sequential prediction tasks [20]. These hybrid architectures are relatively novel in manufacturing research and contribute to expanding the literature on laser cutting prediction.

In addition to DL approaches, several traditional ML models were also employed, including Extreme Gradient Boosting (XGBoost), Random Forest (RF), Support Vector Regression (SVR), and Linear Regression (LR). XGBoost leverages gradient boosting to reduce overfitting and offers insights into parameter importance through feature rankings. The RF model mitigates variance through ensemble learning and allows internal validation via out-of-bag error estimation [21]. SVR was selected for its ability to perform nonlinear regression in high-dimensional feature spaces while remaining resistant to the effects of outliers [22]. LR was included as a baseline model to benchmark the predictive performance of more advanced methods. To thoroughly evaluate the models, a series of analytical techniques was applied. Scatter analysis was used to visualize the relationships between observed and predicted values by plotting them in a Cartesian plane, allowing for the identification of trends, clusters, and outliers. Residual analysis helped to detect systematic errors and inconsistencies by examining the differences between predicted and actual outcomes. A notable strength of this method is its ability to normalize residual distributions across models, enabling direct comparison on a unified scale [23]. The Taylor diagram analysis integrated Standard Deviation (σ), Correlation Coefficient (r), and Root Mean Square Error (RMSE) into a single visual summary, offering a multidimensional framework for assessing the agreement between predicted and observed values across multiple models [24].

**Figure 4:**
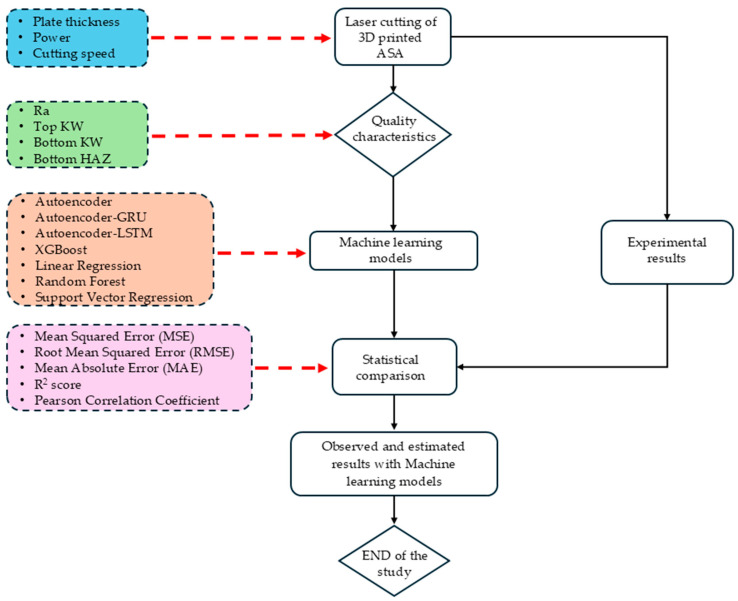
The flowchart of the ML models.

These combined approaches form what are called Multi-Evaluation Methods (MEMs) and, thus, present a very robust framework for conducting a stable and thorough assessment. Unlike single-metric evaluations, MEMs allow one to establish a multidimensional understanding of the model’s behavior and effectiveness. The most important contribution they make to literature is to provide a common, multidimensional evaluation platform for all the models, whether hybrid DL or traditional, in complex situations, such as the laser cutting prediction case. This approach provides a more detailed and objective comparison, aiding superior model selection and process optimization, quantitatively. Model performance was assessed using the standard error metrics Mean Squared Error (MSE), RMSE, Mean Absolute Error (MAE), Coefficient of Determination (R^2^), and Pearson’s correlation coefficients. These were calculated for the test dataset, and an analysis of the comparative results was then conducted to designate the models that were most accurate and had greater generalization ability. This study proposes a variant of hybrid models, such as AE-GRU and AE-LSTM, which are rarely used in experimental manufacturing research, to predict laser cutting performance; this is considered a novel approach. Further, DL methods are compared with the traditional ones and offer a predictive methodology suitable for multi-output optimization in CO_2_ laser cutting processes. Figure 4 presents the flowchart for the ML workflow.

### 3.1. Autoencoder (AE) Model

Autoencoders are neural network models that compress incoming data into a latent representation and then reconstruct it as closely as possible to the original input [25]. These models comprise two main components (encoder—decoder). During the encoding phase, the input $x_{i}$ is transformed by the encoder into a lower-dimensional latent representation $z_{i}$. Equation (1) compresses the input $x_{i}$ into the latent representation $z_{i}$ through the encoder.

(1) $$z_{i}=f\left(x_{i}\right)=\sigma \left(W_{e}\cdot x_{i}+b_{e}\right)$$

where $W_{e}$ represents the encoder weight matrix, $b_{e}$ denotes the encoder bias vector, and σ is the activation function. During the decoding phase, the latent vector $z_{i}$ is processed by the decoder to generate the reconstructed output ${\hat{x}}_{i}$. Equation (2) decodes $z_{i}$ to reconstruct the output ${\hat{x}}_{i}$.

(2) $${\hat{x}}_{i}=g\left(z_{i}\right)=\sigma \left(W_{d}\cdot z_{i}+b_{d}\right)$$

where $W_{d}$ denotes the decoder weight matrix and $b_{d}$ denotes the decoder bias vector.

### 3.2. Long Short-Term Memory (LSTM)

To overcome the ongoing dependency difficulties in RNNs, Hochreiter and Schmidhuber created the LSTM network [26]. In contrast to RNN’s hidden layer, LSTM uses a control unit to retain information. This hidden state is divided into two separate parts: working memory ($h_{t}$) and memory cells ($c_{t}$). The forgetting gate ($f_{t}$) regulates prior sequence memory, whereas $c_{t}$ is in control of sequence feature retention. The output gate ($o_{t}$) controls part of the current memory $c_{t}$, with $h_{t}$ serving as the output. The input gate transfers current input ($x_{t}$) and state ($h_{t-1}$) to memory cells. $H^{\left(L\right)}$ represents an (L)-type (LSTM-specific) hidden state computation function and all internal operations of an LSTM cell: mechanisms of gates (forget, input, output), updates of cell state, and transitions between hidden states. The (L) explicitly differentiates it from other RNN variants (e.g., $H^{\left(G\right)}$ for GRU, $H^{\left(R\right)}$ for RNN). According to Mateus et al., LSTM cells are characterized as follows (Equation (3)) [27].

(3) $$h_{t}^{LSTM}=H^{\left(L\right)}\left(h_{t-1},x_{t},\theta_{LSTM}\right)=\left\{\begin{array}{c} f_{t}=\sigma \left(W_{f}\cdot \left[h_{t-1},x_{t}\right]+b_{f}\right)\;\;\;\;\;\;\\ i_{t}=\sigma \left(W_{i}\cdot \left[h_{t-1},x_{t}\right]+b_{i}\right)\;\;\;\;\;\;\;\\ {\tilde{c}}_{t}=tanh\left(W_{c}\cdot \left[h_{t-1},x_{t}\right]+b_{c}\right)\\ c_{t}=f_{t}⊙c_{t-1}+i_{t}⊙{\tilde{c}}_{t}\;\;\;\;\;\;\;\;\;\;\;\;\\ o_{t}=\sigma \left(W_{o}\cdot \left[h_{t-1},x_{t}\right]+b_{o}\right)\;\;\;\;\;\;\;\\ h_{t}^{LSTM}=o_{t}⊙tanh\left(c_{t}\right)\;\;\;\;\;\;\;\;\;\;\;\;\;\; \end{array}\right.$$

where W refers to weight matrices, b to bias vectors, and σ to the sigmoid activation function. ${\tilde{c}}_{t}$ and $c_{t}$ represent the new and final memory cells. LSTM gates (input, output, and forget) are computed from Equation (3), with the sigmoid function and weight matrices controlling the flow of information through the network, allowing it to learn long-range relationships in sequential data.

### 3.3. Gated Recurrent Unit (GRU)

GRU is a particular type of RNN and an adaptation of LSTM. GRU can solve the issue of gradient vanishing and long-term reliance. It simplifies the LSTM structure and determines the degree of fusion of the present input value with the prior hidden state, as well as the forgetting of the previous state, by incorporating the input and forget gates into an update gate. Compared to LSTM, GRU eliminates one gating unit, resulting in fewer parameters required for training, which can substantially improve training efficiency and significantly reduce calculation resources [28]. Using Equation (4), the update, reset, and cell state gates are calculated in the GRU architecture [27].

(4) $$h_{t}^{GRU}=H^{\left(G\right)}\left(h_{t-1},x_{t},\theta_{GRU}\right)=\left\{\begin{array}{c} r_{t}=\sigma \left(W_{r}\cdot \left[h_{t-1},x_{t}\right]+b_{r}\right)\;\;\;\;\;\;\;\;\;\;\;\;\;\;\;\\ z_{t}=\sigma \left(W_{z}\cdot \left[h_{t-1},x_{t}\right]+b_{z}\right)\;\;\;\;\;\;\;\;\;\;\;\;\;\;\;\\ {\tilde{h}}_{t}=tanh\left(W\cdot \left[{r_{t}⊙h}_{t-1},x_{t}\right]+b\right)\\ h_{t}=\left(1-z_{t}\right)⊙h_{t-1}+z_{t}⊙{\tilde{h}}_{t}\;\;\;\;\;\;\; \end{array}\right.$$

where the weight matrix values for the suitable connected input vector are symbolized by the letters $W_{z}$, $W_{r}$, and W. $b_{r}$, $b_{z}$, and b denote bias. Authors use the symbols σ (logistical sigmoid function), $r_{t}$ (reset gate), $z_{t}$ (updating gate), and ${\tilde{h}}_{t}$ (hidden layer) to indicate candidate layers [29].

### 3.4. Extreme Gradient Boosting (XGBoost) Model

XGBoost was employed to build predictive models for the target variables. During training, it minimizes the regularized objective in Equation (5), which combines the data-fit loss $l\left(y_{pred}^{\left(t\right)},y_{truth}\right)$ with the complexity penalty $\sum_{i}\Omega \left(f_{k}\right)$, ensuring a balance between accuracy and model parsimony [30].

(5) $$L^{\left(t\right)=}\sum_{i}l\left(y_{pred}^{\left(t\right)},y_{truth}\right)+\sum_{i}\Omega \left(f_{k}\right)$$

where $l\left(\cdot \right)$ represents the loss function measuring prediction error and $\Omega \left(f_{k}\right)=\gamma T+\frac{1}{2}\lambda {\left\|\omega \right\|}^{2}$ is a regularization term controlling model complexity, where T is the number of leaves and ω the leaf weights. Separate XGBoost models were trained for each target variable using feature vectors as input, providing a scalable and flexible framework for various prediction tasks [31].

### 3.5. Linear Regression (LR)

For comparison, this study employed a linear regression model; as indicated in Equation (6), it expresses a linear relationship between the dependent variable $\hat{y}$ and the set of independent variables $\left(x_{1},x_{2},\ldots ,x_{m}\right)$ [32].

(6) $$\hat{y}=\beta_{0}+\sum_{j=1}^{m}\beta_{j}x_{j}$$

where $\hat{y}$ is the predicted (dependent) variable, $x_{j}$ is the *j*-th independent variable, $\beta_{j}$ is the regression coefficient for $x_{j}$, and $\beta_{0}$ is the intercept term.

### 3.6. Random Forest (RF) Model

In this study, RF was adopted to construct prediction models. RF is an ensemble approach that trains multiple decision trees on different bootstrap samples and feature subsets, where each tree learns its own prediction function from the input vector $X=\left\{x_{1},x_{2},\ldots ,x_{m}\right\}$. The training dataset is formalized in Equation (7) as $S_{n}=\left\{\left(X_{1},Y_{1}\right),\left(X_{2},Y_{2}\right),\ldots ,\left(X_{n},Y_{n}\right)\right\}$ with $X\in R^{m}$ and, Y∈R. For every bootstrap sample $S_{n}^{\left(b_{i}\right)}$, a separate tree $\hat{f}\left(X,S_{n}^{\left(b\right)}\right)$ is fitted, and the ensemble’s final prediction is obtained by averaging the q tree outputs, as given in Equation (8) [33].

(7) $$S_{n}=\left\{\left(X_{1},Y_{1}\right),\left(X_{2},Y_{2}\right),\ldots ,\left(X_{n},Y_{n}\right)\right\},\;X\in R^{m},\;Y\in R$$

(8) $$\hat{Y}=\frac{1}{q}\sum_{i=1}^{q}{\hat{Y}}_{i}=\frac{1}{q}\sum_{i=1}^{q}\hat{f}\left(X,S_{n}^{\left(b_{i}\right)}\right)$$

where q is the total number of trees, ${\hat{Y}}_{i}$ denotes the output of the *i*-th tree, $S_{n}^{\left(b_{i}\right)}$ represents the *i*-th bootstrap sample, and $\hat{f}\left(X,S_{n}^{\left(b_{i}\right)}\right)$ is the prediction generated by that tree. 

### 3.7. Support Vector Regression (SVR) Model

SVR fits a function that stays within an ε-tube around the targets by minimizing the dual objective in Equation (9) subject to the constraints in Equation (10) [34]. For non-linear patterns, the inputs are implicitly mapped to a higher-dimensional space with the RBF kernel defined in Equation (11), and the learned multipliers $\alpha_{i},\alpha_{i}^{*}$ produce the final regression function $f\left(x\right)=\sum_{i=1}^{n}\left(\alpha_{i}-\alpha_{i}^{*}\right)K\left(x_{i},x_{j}\right)+b$ [35].

(9) $$\underset{\alpha \alpha^{*}}{min}\frac{1}{2}\sum_{i,j=1}^{n}\left(\alpha_{i}-\alpha_{i}^{*}\right)\left(\alpha_{j}-\alpha_{j}^{*}\right)K\left(x_{i},x_{j}\right)+\varepsilon \sum_{i=1}^{n}\alpha_{i}-\alpha_{i}^{*}-\sum_{i=1}^{n}y_{i}\left(\alpha_{i}-\alpha_{i}^{*}\right)$$

(10) $$subject\;to\;\left\{\begin{array}{c} \sum_{i=1}^{n}\alpha_{i}-\alpha_{i}^{*}=0,\\ 0\leq \alpha_{i},\alpha_{i}^{*}\leq C \end{array}\right.$$

(11) $$K\left(x_{i},x_{j}\right)=exp\left(-\gamma {\left\|x_{i}-x_{j}\right\|}^{2}\right)$$

where C is the regularization (penalty) parameter, $\alpha_{i}$ and $\alpha_{i}^{*}$ are the Lagrange multipliers, and $K\left(x_{i},x_{j}\right)$ denotes the kernel function. The vector $x_{i}$ represents the *i*-th input (feature) in the training dataset. The bias term b is obtained from the Karush–Kuhn–Tucker (KKT) conditions [36].

### 3.8. Evaluation Metrics

In ML and statistics, various evaluation metrics are used to assess model performance. MSE is an evaluation metric that measures the average of the squared differences between actual and predicted values in ML models. Lower MSE values indicate a better-fitting model [37]. It is sensitive to the magnitude of errors and is computed with the help of Equation (12).

(12) $$MSE=\frac{1}{n}\sum_{i=1}^{n}{\left(y_{i}-{\hat{y}}_{i}\right)}^{2}$$

RMSE is frequently used in the prediction of ML models to calculate the distance between predicted and actual values (Equation (13)). It is also a quadratic metric that measures the magnitude of the error [37].

(13) $$RMSE=\sqrt{\frac{1}{n}\sum_{i=1}^{n}{\left(y_{i}-{\hat{y}}_{i}\right)}^{2}}$$

MAE is defined as the average of the absolute errors between the true and predicted values of observations; it has a clear interpretation and is not affected by extreme values. An analytical expression for MAE is provided in Equation (14).

(14) $$MAE=\frac{1}{n}\sum_{i=1}^{n}\left|y_{i}-{\hat{y}}_{i}\right|$$

The coefficient of determination (R^2^) reflects how well the model explains the variance in the dependent variable, with values approaching 1 signifying a stronger fit (Equation (15)) [37].

(15) $$R^{2}=1-\frac{\sum_{i=1}^{n}{\left(y_{i}-{\hat{y}}_{i}\right)}^{2}}{\sum_{i=1}^{n}{\left(y_{i}-\bar{y}\right)}^{2}}$$

As shown in Equation (16), Pearson’s Correlation Coefficient quantifies the strength of the linear relationship between predicted and actual values. It ranges from −1 to +1, with values near +1 indicating a strong positive correlation.

(16) $$Pearson=\frac{\sum_{i=1}^{n}\left(y_{i}-\bar{y}\right)\left({\hat{y}}_{i}-\bar{\hat{y}}\right)}{\sqrt{\sum_{i=1}^{n}{\left(y_{i}-\bar{y}\right)}^{2}}\sqrt{\sum_{i=1}^{n}{\left({\hat{y}}_{i}-\bar{\hat{y}}\right)}^{2}}}$$

In this content, $y_{i}$ and ${\hat{y}}_{i}$ are the actual and predicted values of the *i*-th sample, respectively, $\bar{y}$ and $\bar{\hat{y}}$ are the averages of the actual and predicted values, respectively, and n indicates the overall number of specimens used.

## 4. Results and Discussion

### 4.1. Evaluation of Cutting Performance

The following section presents an interpretation of the laser cutting performance indicators derived from CO_2_ laser cutting experiments to assess the cutting quality of the 3D-printed ASA materials. In this context, the following results were analyzed in detail: (1) Ra for surface quality; (2) Top Kerf Width and (3) Bottom Kerf Width for kerf geometry; (4) Bottom Heat-Affected Zone for its behavior under heat influence.

#### 4.1.1. Surface Roughness

**Figure 5:**
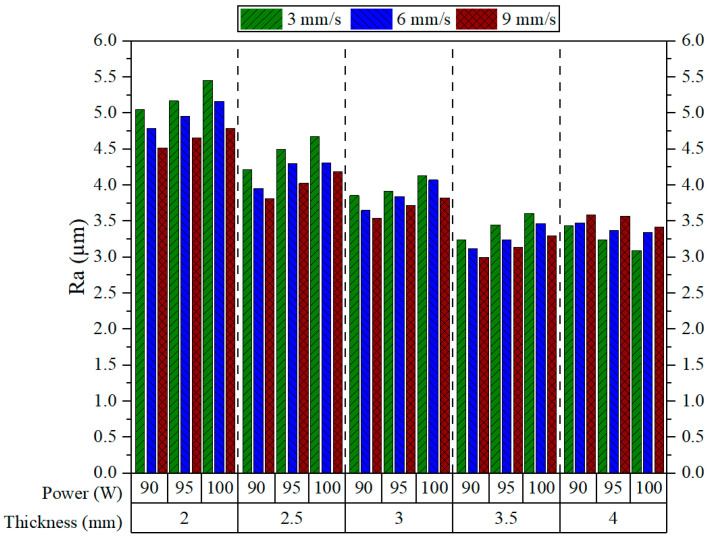
Ra results.

Figure 5 shows the average Ra values measured for different combinations of cutting parameters. Each group of bars represents a specific material thickness and shows the effects of the power–speed combinations. Generally, an increase in material thickness decreases Ra. However, this trend is broken when the thickness increases from 3.5 to 4 mm, where there is a sudden increase in Ra; other than this, Ra decreases with increasing cutting speed, except at 4 mm thickness, where the opposite happens. Similar results were obtained by Kechagias et al., who reported a reduction in Ra values with increased cutting speed [7]. Similarly, increasing the laser power generally leads to a rise in Ra across all thicknesses, except at 4 mm, where higher power results in a decrease in Ra, exhibiting an opposite pattern to that seen with cutting speed. The same study by Kechagias et al. also revealed that Ra increases with increasing power [7]. With the thinnest plates (2 mm), Ra values are generally high and exceed 5.0 µm.

The maximum Ra was registered at a speed of 3 mm/s and a power of 100 W (5.454 µm). These high values are likely to be the result of excess thermal accumulation and insufficient energy for the process of stable material removal [38]. As the thickness increases to 2.5 mm and 3 mm, Ra values begin to decrease somewhat. This means that thicker plates provide room for better thermal diffusion to improve melt flow and reduce surface irregularities. The lowest Ra value is reported at 3.5 mm thickness, 90 W power, and 9 mm/s speed (2.998 µm), indicating a proper balance of energy input and heat distribution at the thickness. However, a slight increase in Ra is observed at 4 mm, which may be attributed to either partial melt ejection or thermal accumulation at greater depths. Overall, higher cutting speeds (9 mm/s) combined with lower laser powers consistently resulted in lower Ra across all thicknesses—except at 4 mm—thereby making this combination optimal for enhanced cut quality.

**Figure 6:**
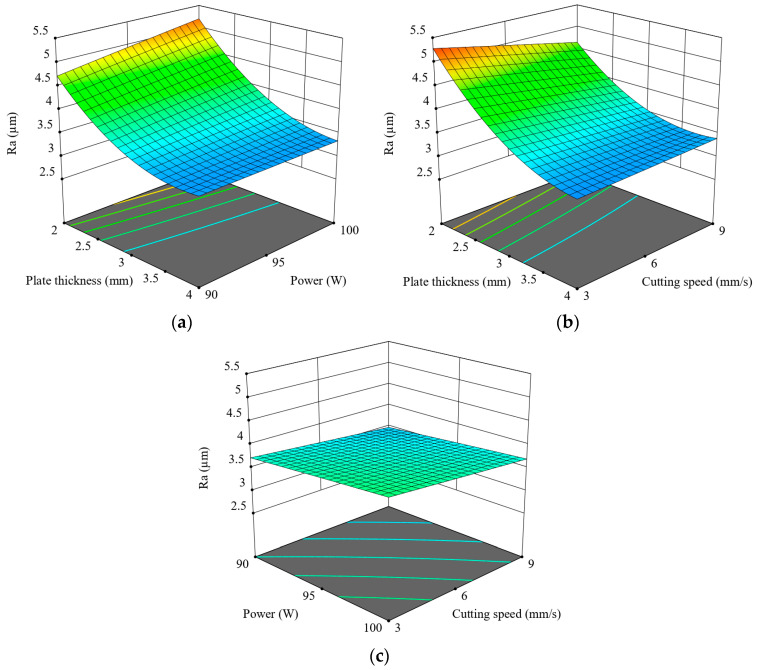
Three-dimensional graphics for Ra: (**a**) p-t, (**b**) v-t, (**c**) v-p.

The 3D response surface plots illustrate the combined effects of the process parameters on Ra (Figure 6). Figure 6a, which shows the interaction between plate thickness and laser power, shows a clear decreasing trend in Ra as both thickness and power increase, up to 4 mm. Thin plates processed at lower power levels tend to yield lower Ra values compared to those cut at higher powers. This indicates that an adequate amount of laser energy is crucial to ensure proper melting and effective ejection of material, especially for thicker sections. Figure 6b, which maps the relationship between plate thickness and cutting speed, reveals a similar pattern. Higher cutting speeds substantially reduce Ra, although this trend is again disrupted at 4 mm thickness. This phenomenon can be attributed to the shorter interaction time between the laser beam and the material surface at higher speeds, which restricts heat buildup and reduces surface damage. The obtained result is consistent with the findings reported by Moradi [39]. The smoothest regions are observed at the highest speed and moderate-to-high thickness combinations.

**Figure 7:**
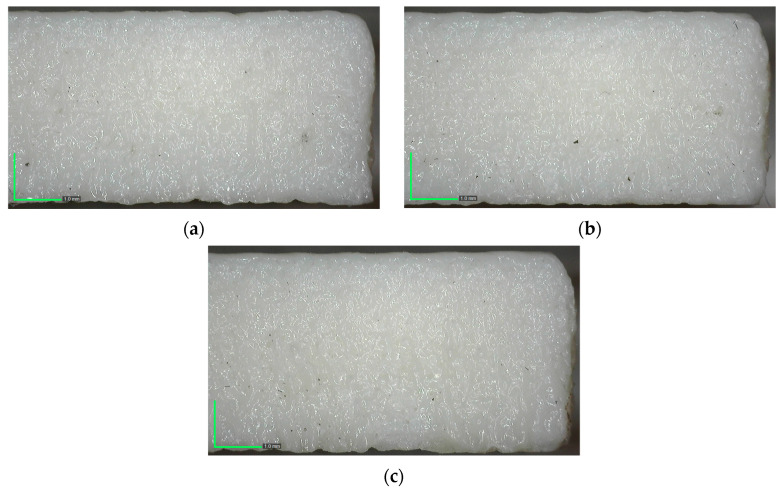
Surface images of specimens cut at varying speeds: (**a**) 3 mm/s, (**b**) 6 mm/s, (**c**) 9 mm/s.

Figure 6c depicts the relationship between laser power and cutting speed under constant plate thickness conditions. Compared to the other plots, this surface appears relatively flat, suggesting that the combined effect of power and speed is less significant than their individual interactions with material thickness. Nevertheless, lower Ra values are still achieved at lower laser power and cutting speed combinations. Overall, the surface plots confirm that plate thickness is the most influential parameter affecting Ra, particularly through its interaction with power and speed. The optimal condition for minimizing Ra is achieved with a 3.5 mm plate thickness, 90 W power, and 9 mm/s speed, as supported by both experimental results and surface modeling analyses. Figure 7 shows how cutting speed affects surface morphology under constant conditions of 90 W power and thickness of 3.5 mm, demonstrating that higher speeds—from 3 mm/s to 9 mm/s—result in noticeably smoother and more uniform surfaces.

#### 4.1.2. Top Kerf Width

**Figure 8:**
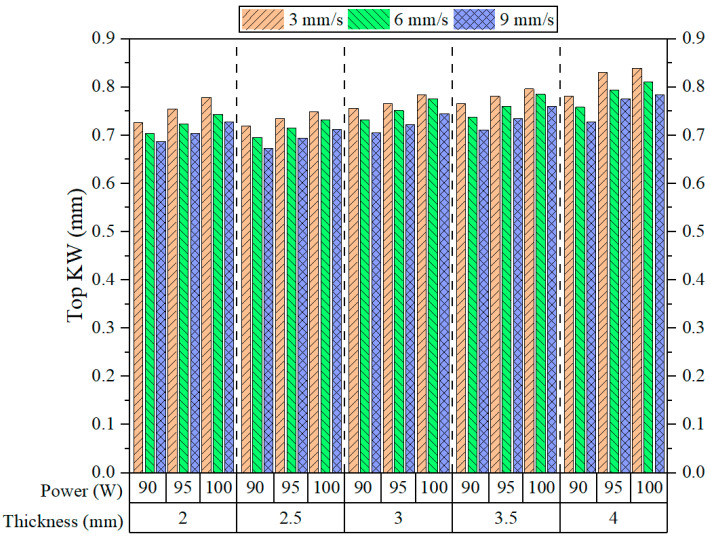
Top KW results.

Figure 8 presents the experimental Top KW results, revealing consistent trends across different levels of power, speed, and thickness. Overall, it can be observed that Top KW generally declines as speed increases, independent of the applied power or thickness. This observation corroborates Sabri et al.’s findings [14]. This phenomenon is attributed to the reduced interaction time between the laser beam and the polymer surface at high cutting speeds. As a result, lateral heat diffusion and melting beyond the immediate cutting line are minimized. For all examined thickness levels, the highest Top KW values consistently occur at the lowest speed of 3 mm/s. In contrast, lower values are typically observed when the speed increases to 9 mm/s. This behavior becomes more evident with increasing material thickness, implying a stronger effect of cutting speed on KW as material volume increases. For instance, looking at 4 mm thickness, the difference in Top KW between the cutting speeds of 3 and 9 mm/s appears far greater than for a thinner plate.

Concerning laser power, an increase from 90 W to 100 W generally results in a slight increase in the Top KW for most thicknesses and cutting speeds. This result is consistent with the observations reported by Kechagias et al. [40]. The increase in KW has been explained to be a function of the energy input: as energy input increases, more thermal degradation and melting of materials occurs, thus creating a wider kerf on the surface [41]. However, this increase is not uniform and becomes less apparent as the cutting speed is increased; most probably, the reduction in interaction time counteracts the effect of increased power. Based on the observation of the material thickness dependence of Top KW values, they tend to increase progressively as the thickness increases from 2 mm to 4 mm. It is only when the transition from 2 mm to 2.5 mm thickness occurs that a decrease in Top KW is noted. Then, when the thickness is changed from 2.5 mm to 3 mm, a sharp increase in values is again noted. A thick material requires more energy for penetration, leading to an increase in heat accumulation on the upper surface and, hence, wider kerf openings [42]. However, this increase in rate is not particularly remarkable and is more visible at a low speed and high power, where the thermal input is concentrated.

**Figure 9:**
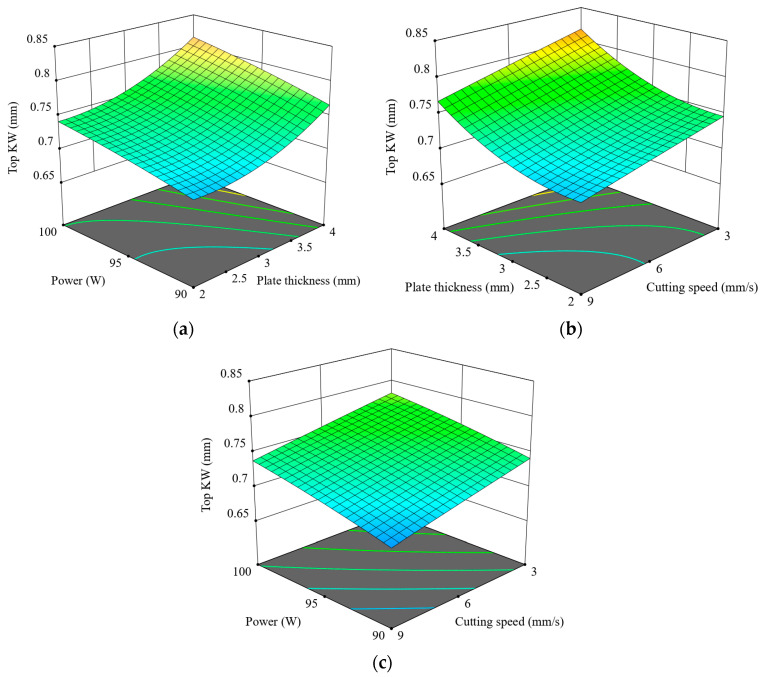
Three-dimensional graphics for Top KW: (**a**) t-p, (**b**) v-t, (**c**) v-p.

**Figure 10:**
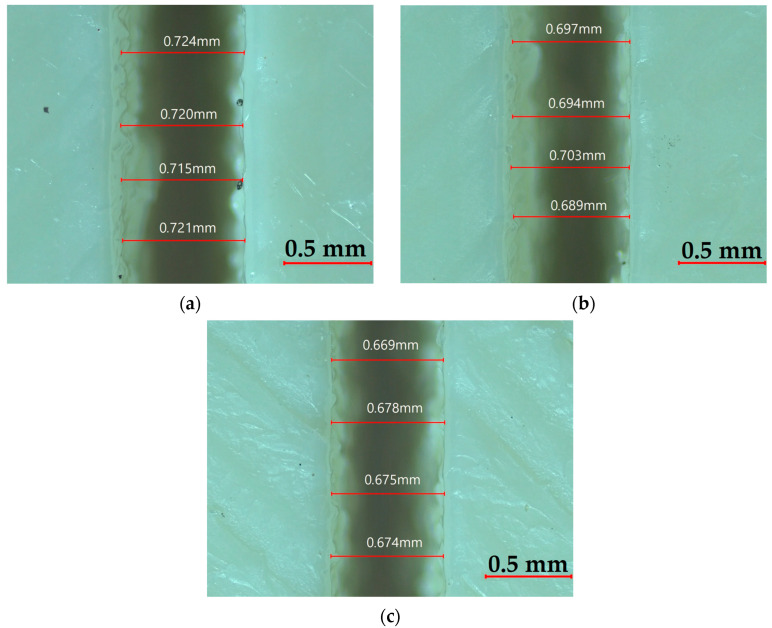
Top KW at varying speeds: (**a**) 3 mm/s, (**b**) 6 mm/s, (**c**) 9 mm/s.

Figure 9 illustrates the combined influence of power, speed, and thickness on the Top KW. In Figure 9a, Top KW increases gradually with an increase in thickness and power, especially at 4 mm and 100 W, owing to the higher energy input and consequent thermal accumulation. As the cutting speed increases in Figure 9b, there is a marked reduction in Top KW, especially at higher thickness values, due to shorter interaction times limiting heat diffusion. A relatively minor interaction between power and speed is observed in Figure 9c, indicating that their effect on Top KW is not particularly significant when thickness is held constant. Low power and high speed, however, consistently yield the minimum Top KW. Of the three parameters, plate thickness plays the most consequential role. It can be concluded that the Top KW can be minimized when subjected to lower power, higher cutting speed, and intermediate material thickness. The surface plots concur with experimental data, emphasizing the thermal dynamics controlling kerf formation. Figure 10 shows microscopic images of the Top KW for 2.5 mm thick ASA plates cut at 90 W. An increase in speed from 3 mm/s to 9 mm/s results in a noticeable decrease in KW, indicating that higher cutting speeds enhance dimensional accuracy.

#### 4.1.3. Bottom Kerf Width

**Figure 11:**
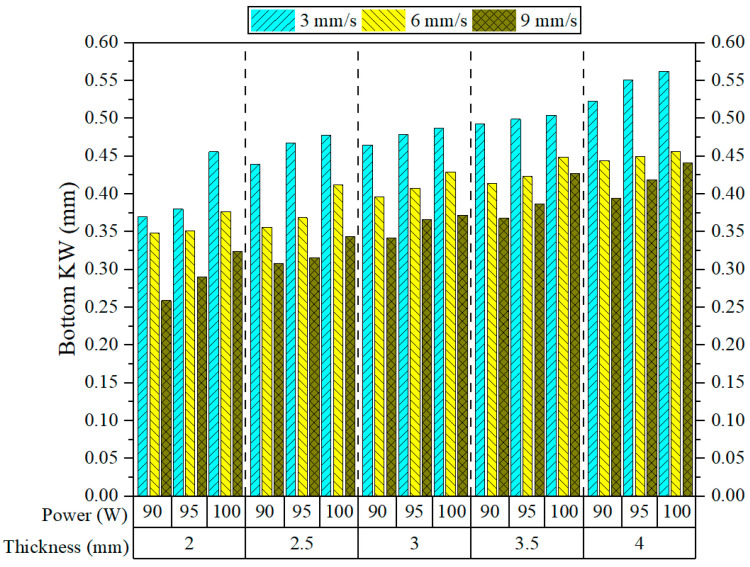
Bottom KW results.

Figure 11 demonstrates the range of Bottom KW measurements for 3D-printed ASA parts processed by CO_2_ laser cutting, alongside the corresponding machining parameters, including power, speed, and thickness. Looking at the graph, it is significant to note that the speed seems to dominate in determining Bottom KW. As it goes on increasing from 3 mm/s to 9 mm/s, irrespective of any power, and thickness, there is a decrease in Bottom KW systematically. This trend has been reported by Sabri et al. [14]. This trend can be explained by the reduced interaction time between the laser beam and the material at higher cutting speeds. As a result, thermal diffusion becomes limited, leading to the formation of a narrower kerf at the bottom surface. Bottom KW values are always highest at a low speed of 3 mm/s, especially for thick plates. At a thickness of 4 mm and a power of 100 W, for example, Bottom KW was recorded to be above 0.55 mm, which is the widest kerf measured in this study. Conversely, the narrowest kerfs—less than 0.35 mm—were obtained at 9 mm/s and 90 W for the relatively thin plates of 2 and 2.5 mm. Thus, it can be concluded that combinations of high speeds and low power levels give effective results for reducing bottom-side heat buildup and excessive melting. Similar results have been reported in the literature by other researchers [38,40].

In CO_2_ laser cutting of thermoplastics, high cutting speeds at low laser power must be used to limit bottom-side heat buildup and melt enlargement. It serves to shrink laser weld HAZ and, in the process, improve cutting quality, making the process versatile over a wide range of thermoplastic materials. The effect of power is less prominent than that of laser speed, but it is nonetheless to be monitored. An increase in power from 90 W to 100 W causes a slight yet consistent increase in Bottom KW for all material thicknesses, especially at low cutting speeds. This is understood, as higher laser power translates into more thermal energy, enlarging the melt pool and kerf [43]. Conversely, at high cutting speeds, this effect is less visible because of the shorter interaction time limiting thermal penetration toward the lower surface. From the perspective of material thickness, Bottom KW is prone to increase as the thickness increases. Thus, as thickness is increased, it requires more energy to pass through, and the extra thermal energy is used to move downward, leading to kerf widening at the bottom. The bottom KW showed a definite rising trend with increasing thickness from 2 mm to 4 mm, especially at low speeds and high-power levels. The biggest jump in KW occurred between 3.5 mm and 4 mm thickness; this could be attributed to factors such as higher thermal buildup, less efficient melt ejection, and uneven heat distribution. Hence, all in all, cutting speed emerges as the most effective factor affecting Bottom KW, followed in importance by material thickness and laser power.

**Figure 12:**
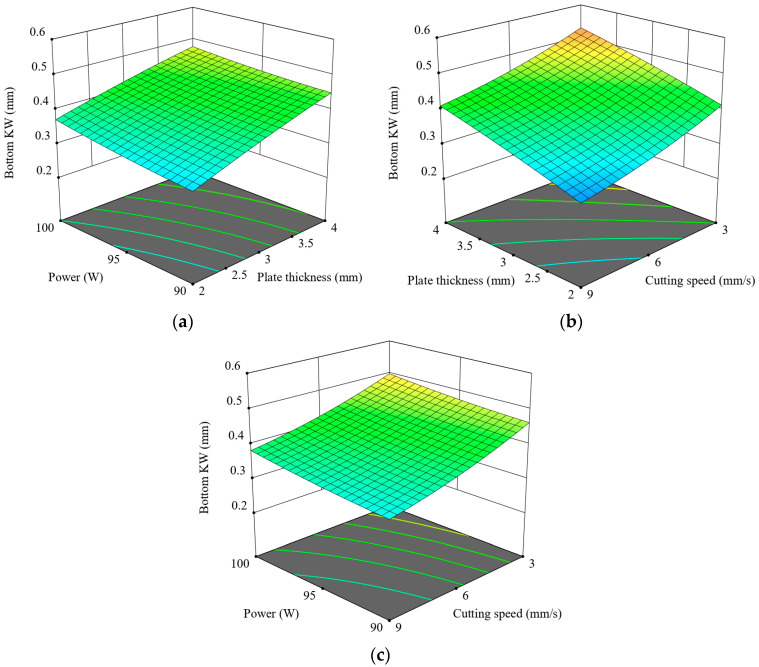
Three-dimensional graphics for Bottom KW: (**a**) t-p, (**b**) v-t, (**c**) v-p.

**Figure 13:**
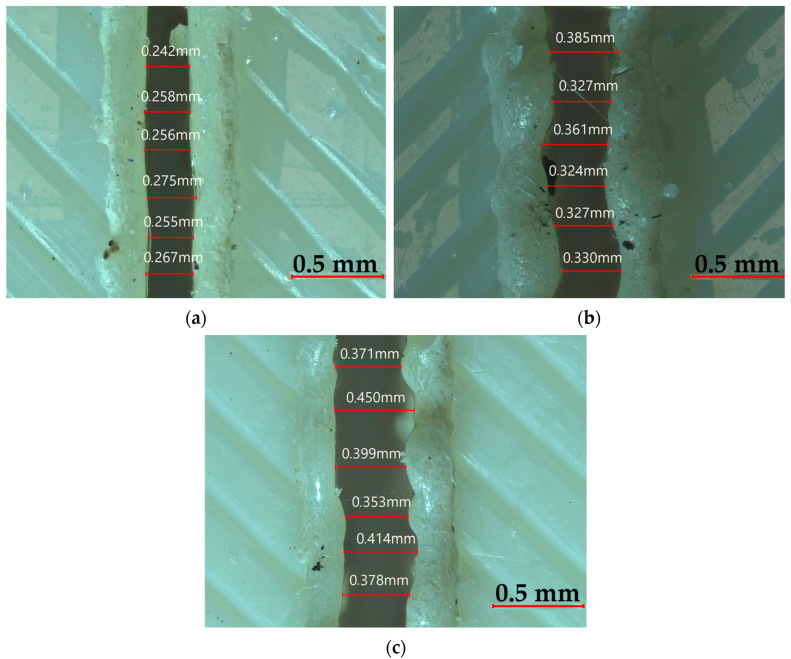
Bottom KW at varying thicknesses: (**a**) 2 mm, (**b**) 3 mm, and (**c**) 4 mm.

The impact of laser cutting parameters on Bottom KW is depicted in Figure 12. Figure 12a shows Bottom KW rising with greater thickness and power, as thicker materials and higher power levels produce wider kerf formations at the bottom due to excessive heat accumulation. As shown in Figure 12b, Bottom KW increases with thickness and lower speeds, implying longer interaction times between laser and material, resulting in deeper thermal penetration and wider bottom kerfs. Figure 12c exhibits a more influential effect of cutting speed compared to power, considering that Bottom KW reduces notably with rising speed, particularly at lower power. This is because the latter restricts the time for heat diffusion and the melting depth due to limited interaction time at high speeds. Notably, the lowest Bottom KW is always achieved at the combination of the lowest power and the fastest cutting speed (90 W–9 mm/s). In contrast, the largest KW is observed at 100 W and 3 mm/s, particularly for thick plates. In summary, plate thickness is the variable with the greatest effect, followed by cutting speed and then laser power, which has a moderate effect on Bottom KW. Figure 13 illustrates the changes in Bottom KW under constant conditions of 9 mm/s speed and 90 W power. As the thickness increases, Bottom KW also increases, with the narrowest kerf observed at 2 mm and the widest at 4 mm, likely due to deeper heat penetration and increased material melting.

#### 4.1.4. Bottom Heat-Affected Zone

**Figure 14:**
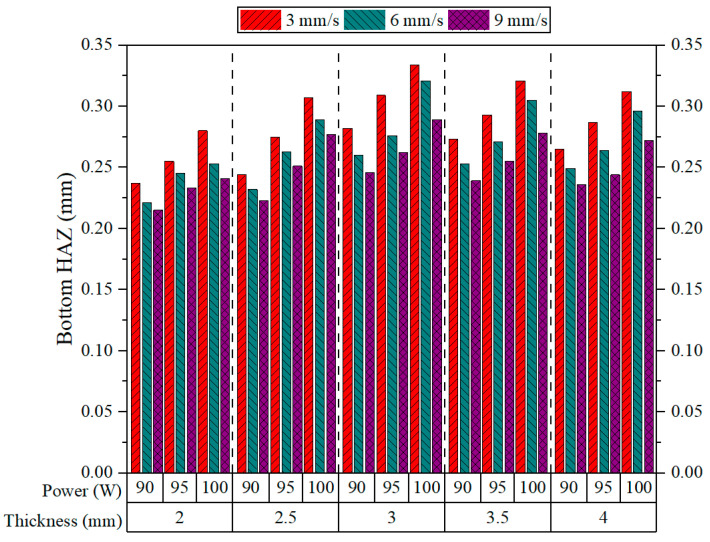
Bottom HAZ results.

Figure 14 depicts the Bottom HAZ variation among different plate thicknesses at various laser powers and cutting speeds operating under CO_2_ laser cutting. There seems to be a general trend wherein Bottom HAZ increases with decreasing cutting speed, irrespective of thickness or power. This trend was also observed by Sabri et al., who found that an increase in cutting speed results in a reduction in the HAZ [14]. This type of behavior is attributed to the longer interaction time of the laser beam with the material at a lower speed, which allows for more thermal penetration and heat accumulation in the bottom region of the cut. The highest Bottom HAZ values are always noted at 3 mm/s across all speed levels, while the lowest values are often observed at 9 mm/s. The theory that faster cutting speeds reduces the depth of thermal diffusion is also supported by these observations. At 3 mm thickness and 100 W power, for example, Bottom HAZ exceeds 0.33 mm at 3 mm/s and drops to less than 0.27 mm at 9 mm/s. This is a strong endorsement for the importance of interaction time in dictating the amount of thermal damage to the bottom regions of the material.

Of the 90 W and 100 W power levels, the Bottom HAZ rises as the power is increased for every thickness and speed analyzed. This result is supported by the works of Der et al. [42,44]. The higher the power level, the greater the thermal energy transferred to the material, which favors a larger heat-affected zone’s width and depth. Whereas the effect of laser power manifests strongly with lower cutting speeds, the simultaneous high energy entries and longer exposure times enhance more thermal gradients. The thickness of the material also plays an important role in the formation of Bottom HAZ [45]. As the thickness changes from 2 mm to 3 mm, the values of HAZ tend to increase, especially at low cutting speeds and high-power levels. However, an increment in thickness from 3 mm to 3.5 mm results in a reduction in Bottom HAZ, and this declining trend continues until a thickness of 4 mm is reached. This is due to more efficient heat dissipation or less efficient energy transfer in thicker sections. Generally, Bottom HAZ tends to be minimized under the combined effects of high speed, low power, and thin plate sections (2–2.5 mm). These settings result in the accumulation of less heat and limit the thermal damage to the bottom surface depth, rendering an optimum process zone for cutting ASA materials with a CO_2_ laser at minimum HAZ.

**Figure 15:**
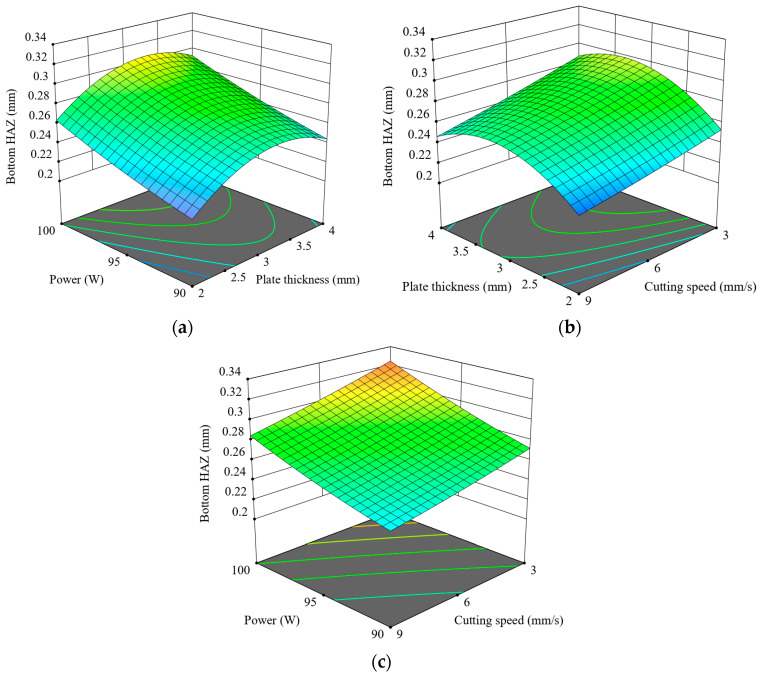
Three-dimensional graphics for Bottom HAZ: (**a**) t-p, (**b**) v-t, (**c**) v-p.

Figure 15 presents the interactions between the cutting parameters and Bottom HAZs during CO_2_ laser cutting. In Figure 15a, Bottom HAZ widens as the power and thickness increase, peaking between about 95 and 100 W and a thickness of 3 to 3.5 mm, suggesting maximum thermal accumulation under these conditions. Figure 15b displays the same trend: the Bottom HAZ increases with thickness and decreases with speed, thereby emphasizing long interaction time for heat diffusion. However, the thickest plates at the slowest speed (3 mm/s) result in the greatest HAZ depth. Figure 15c confirms that when the power is high and the speed is low, the Bottom HAZ increases to a maximum, supporting the mechanism where high energy input combined with prolonged exposure leads to significant thermal damage.

**Figure 16:**
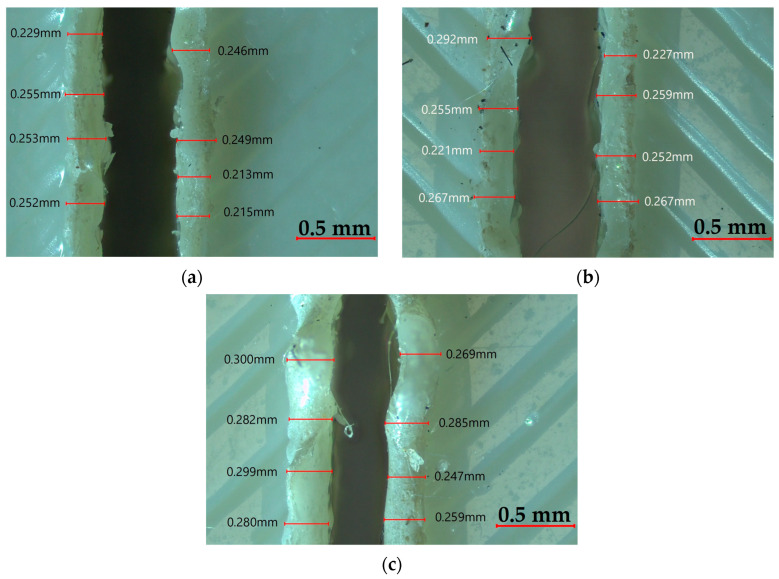
Bottom HAZ at varying powers: (**a**) 90 W, (**b**) 95 W, (**c**) 100 W.

In all three plots, the Bottom HAZ is always lowest at low power (90 W) and high speed (9 mm/s), especially for thinner materials. These results highlight speed as the most influential parameter for reducing HAZ, followed by thickness and then laser power [46]. Hence, optimum cutting can be achieved by lowering the power while working with thin plates at higher speeds, so that heat penetration is kept at a minimum. Figure 16 shows the Bottom HAZ of 3.5 mm thick ASA plates cut at 9 mm/s, where increasing power from 90 W to 100 W leads to a noticeable enlargement of the HAZ, indicating greater thermal diffusion and potential material degradation at higher energy input levels.

### 4.2. ANOVA-Based Findings

Table 2 presents the ANOVA results, explaining the statistical significance and contribution of the selected process parameters on the critical laser cutting quality characteristics of 3D-printed ASA parts. In the table, plate thickness is denoted as t, laser power as p, cutting speed as v, error as E, and total as T. Plate thickness is the most dominant factor affecting Ra values, accounting for about 89% of the total variation. This high influence is supported by a very large F-value of 136.11 and a *p*-value of less than 0.001, proving the statistical importance of this effect. Laser power (2.30%) and cutting speed (2.81%) contribute to a much lesser degree, but they are also associated with *p*-values of 0.003 and 0.001. The model for Ra performs well in prediction, with R^2^ = 94.11%, R^2^ (adj) = 92.81%, and R^2^ (pred) = 90.80%, implying that the model considered could explain many of the variations in Ra responses.

All three inputs significantly affect the Top KW, with plate thickness exerting the most influence at 49.26%. Cutting speed and laser power account for 25.95% and 22.31% of the effect, respectively. These F-values are very high for the three factors, i.e., cutting speed (188.44), plate thickness (178.88), and power (162.04), with *p*-values smaller than 0.001, confirming the strength of these relationships. As the error is small (2.48%) and the model fit statistics are very strong (R^2^ = 97.52%, R^2^ (adj) = 96.97%, R^2^ (pred) = 96.13%), the adequacy and prediction capability of the model are excellent, indicating that all variables significantly influence Top KW, with plate thickness being the most dominant parameter in controlling kerf formation on the upper surface of ASA parts. Regarding Bottom KW, cutting speed becomes the dominant factor, accounting for 52.76% of the total variation. Next, plate thickness contributes 37.88%, followed by laser power with just 5.90%. The F-value for cutting speed is very high at 274.45, highlighting a very strong effect. Thus, increased cutting speed remarkably reduces bottom KW owing to decreased laser–material interaction time. For all three factors, the *p*-values are less than 0.001, thus confirming statistical significance. With a 3.46% error and an R^2^ of 96.54%, the regression model for Bottom KW is robust and reliable.

In process optimization for minimizing kerf widening at the bottom of the cut, these results support the prioritization of speed. For Bottom HAZ, power takes the crown as the most influential factor, accounting for 44.75% of the total variance, followed by thickness (27.30%) and speed (23.97%). With all three parameters boasting strong statistical significance, *p*-values less than 0.001, and high F-values (power: 206.24, speed: 108.41), the results are proof that increasing power levels increase thermal penetration and thus wider HAZ zones, particularly when combined with the lowest speeds. Model error remained at a low 3.98%, and the regression model showed great explanatory and predictive power: R^2^ = 96.02%, R^2^ (adj) = 95.14%, and R^2^ (pred) = 93.78%. Overall, the ANOVA results reveal a quality metric dominated by a different factor.

### 4.3. ML Modeling Results

The experimental dataset comprises the independent variables thickness (mm), power (W), and speed (mm/s), as well as the dependent variables Ra (µm), Top KW (mm), Bottom KW (mm), and Bottom HAZ (mm). The data were randomly partitioned into 80% for training and 20% for testing. The Autoencoder (AE) model features a streamlined architecture beginning with a 3-dimensional input layer, followed by a fully connected encoder layer containing 16 neurons with ReLU activation, and concluding with a single-output decoder layer. This model was trained using MSE as the loss function and optimized with the Adam algorithm at a learning rate ($l_{r}$) of 0.001. Building upon this foundation, the Autoencoder–GRU (AE-GRU) variant incorporates a 16-unit GRU layer as its encoder component, processing the input data sequentially while maintaining the same optimization parameters through 100 training epochs. The Autoencoder–LSTM model similarly employs a 16-neuron LSTM layer to encode temporal patterns, complemented by a time-distributed output layer, with training parameters consistent with the GRU variant to ensure comparable optimization conditions.

The modeling approach makes a deep blend of feature representation learned by deep neural networks and computational efficiency characteristics of traditional ML algorithms, thereby producing a strong structure for the comparative analysis of laser cutting parameter predictor performance [47]. Each architecture was selected given its unique advantages in characterizing nonlinear relationships and dependencies that can be found in manufacturing process data. The methods of ensembles include XGBoost regression with estimators set to 100 and learning rates set to 0.1, which have built-in mechanisms for regularization to prevent overfitting. The LR model, using ordinary least squares estimation, is implemented for comparison purposes. RFs consist of 100 decision trees with default settings. SVR is set with an RBF kernel with hyperparameters tuned to C = 100 and gamma = 0.1 to maximize its performance. The LR method estimates the relationship between inputs and outputs using the least squares method. All the computational processing in estimating computational implementation was executed on a laptop configured as follows: processor Intel Core i7-12700H @ 4.70 GHz, RAM 32 GB DDR4, and GPU NVIDIA GeForce RTX 3050 (6 GB).

All computational procedures and visualizations were performed using Python (v3.11.13) within a unified software environment including Pandas (v2.2.2), NumPy (v2.0.2), scikit-learn (v1.6.1), TensorFlow (v2.18.0), XGBoost (v2.1.4), and Matplotlib (v3.10.0). The experimental framework consisted of fixed training parameters for all DL algorithms; hence, every architecture was trained for exactly 100 epochs for an intact and fair comparison. Neural networks were optimized with a single methodology of using Adam optimizers throughout all DL implementations to prevent different ways of stochastic gradient descent while pursuing comparable learning conditions [48]. Traditional ML models were implemented using their standard library versions, with careful attention given to hyperparameter tuning to optimize predictive performance for the specific application. All model architectures, particularly the neural network designs, were carefully dimensioned to effectively process the 3-dimensional input parameter space of the laser cutting dataset, ensuring appropriate capacity for feature extraction while avoiding unnecessary complexity [49]. This systematic approach to model configuration and training enabled meaningful comparisons between different algorithmic strategies while maintaining optimal performance for each method. All traditional models were executed using their standard scikit-learn implementations with default parameters unless otherwise specified, ensuring reproducibility while maintaining competitive performance benchmarks [50].

In the case of the work conducted in this study, two types of Autoencoder models are designed for sequence data: i.e., Autoencoder–GRU and Autoencoder–LSTM, depending on the type of units used in their architecture, GRU units and LSTM units, respectively. Both work on a single sample with a shape of (1, 3). The input is passed to the encoder formed by 16 GRU or LSTM units, using ReLU activation, then to a Repeat Vector Layer (RVL) that replicates the encoded context along time steps. The context is then decoded through either a GRU or LSTM layer with three units and ReLU activation, and finally through a Time Distributed Dense Layer (TDDL) to produce output at each time step. The two models were trained for 100 epochs with a batch size of eight. The XGBoost algorithm was set up as a gradient boosting method with 100 estimators and a learning rate ($l_{r}$) value of 0.1 while a fixed random state of 42 was set for the sake of reproducibility. All the model parameters and summary architecture details are given in Table 3.

**Figure 17:**
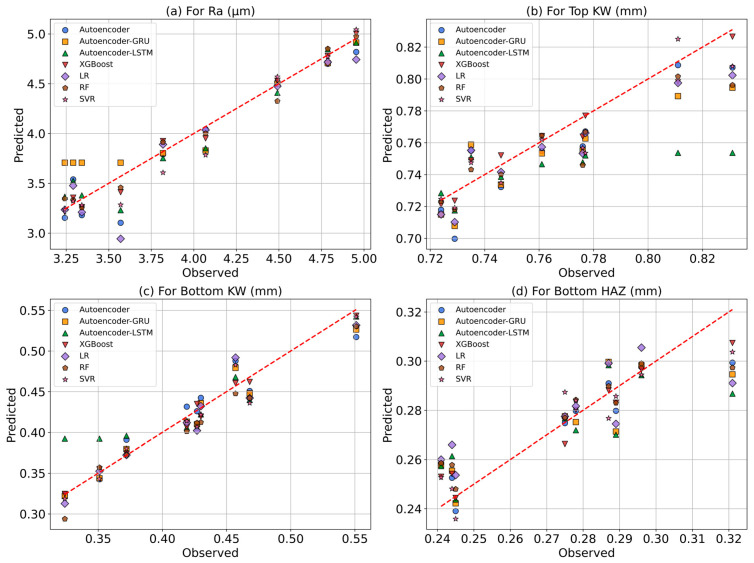
Predicted–observed graphs: (**a**) Ra, (**b**) Top KW, (**c**) Bottom KW, (**d**) Bottom HAZ.

Figure 17 compares the predicted values obtained from seven different ML models with the actual observed values for each response. The red dashed line represents the ideal equality line (y = x), offering a visual means to evaluate the accuracy of model predictions. Among the ML models developed for predicting Ra, XGBoost, RF, and Autoencoder models show particularly high agreement with the observed values and cluster closely around the equality line. These models have successfully captured the complex relationships between the laser cutting parameters affecting Ra. However, some deviations are observed in the Autoencoder–GRU and LR models, which may indicate insufficient learning performance. For Top KW, greater deviations in prediction performance are seen compared to Ra, especially in the Autoencoder–LSTM model. Noticeable discrepancies from the observed values are evident in both the Autoencoder–LSTM and Autoencoder–GRU models. Nonetheless, the XGBoost, RF, and SVR models produced relatively accurate results.

In the case of Bottom KW, all models—except for Autoencoder–LSTM—demonstrate generally high performance. The predicted values from the XGBoost and SVR models are very close to the actual values. Furthermore, in the Bottom KW range between 0.40 and 0.45 mm, all models are observed to cluster densely along the ideal equality line. For Bottom HAZ prediction, the XGBoost model emerged as the most successful, generating results closest to the reference line with the best fit coefficients and the lowest prediction errors. However, it shows the lowest prediction performance among all responses. Unlike the general trend, Bottom HAZ values above 0.32 mm tend to fall below the ideal equality line (y = x). The LR and Autoencoder–GRU models produce more scattered predictions and show lower accuracy compared to the other models. Overall, these scatter plots reveal that the XGBoost model achieves high prediction accuracy across all responses and demonstrates strong agreement with the experimental data. These findings support the statistical evaluations presented in previous sections and confirm that these models are effective tools for predicting multiple outputs in CO_2_ laser cutting applications.

**Figure 18:**
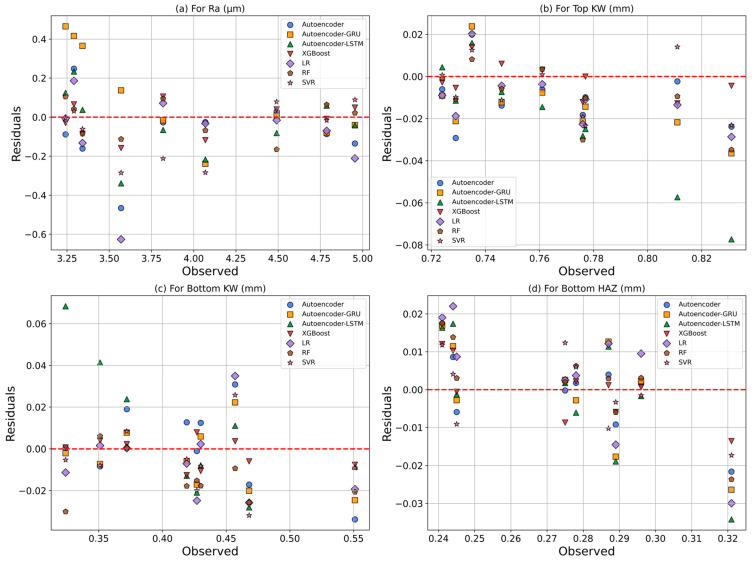
Residual graphs: (**a**) Ra, (**b**) Top KW, (**c**) Bottom KW, (**d**) Bottom HAZ.

Residual analyses of observed and predicted values for each response metric are illustrated in Figure 18. In the prediction of Ra, the XGBoost and RF models demonstrated high consistency, as indicated by the balanced distribution of residuals around zero. In contrast, the Autoencoder–GRU model exhibited the highest positive deviation above the zero line, while the LR model showed the most significant negative deviation below it. For the prediction of Top KW, the XGBoost model, followed by the RF model, achieved the lowest errors and the highest coefficients of determination. Conversely, the Autoencoder–LSTM model produced greater uncertainty in predictions, as reflected by its wider range of deviations. In the prediction of Bottom KW, the XGBoost model yielded predictions closest to the zero line, characterized by the lowest residuals and the highest goodness of fit. The Autoencoder–LSTM model, on the other hand, displayed a more dispersed residual pattern, especially in the positive direction, resulting in lower accuracy compared to other models. Regarding Bottom HAZ prediction, the XGBoost and RF models again stood out, with the lowest prediction errors and the most balanced residual distribution around zero. In contrast, the LR model exhibited broader and more systematic deviations, indicating poor predictive performance. Overall, the residual analyses highlight that the XGBoost model consistently produced narrowly and symmetrically distributed residuals around zero, demonstrating the most stable and reliable predictive performance. In comparison, the Autoencoder–GRU for Ra, the Autoencoder–LSTM for Top KW and Bottom KW, and the LR model for Bottom HAZ showed systematic deviations and broader residual distributions, indicating a lack of capability in capturing complex, nonlinear relationships.

**Figure 19:**
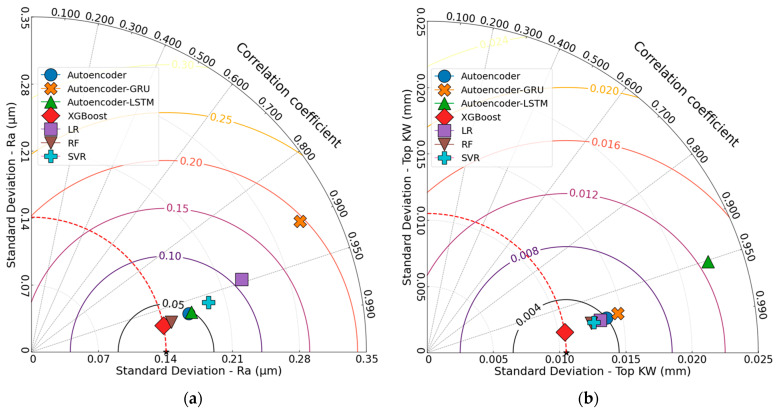

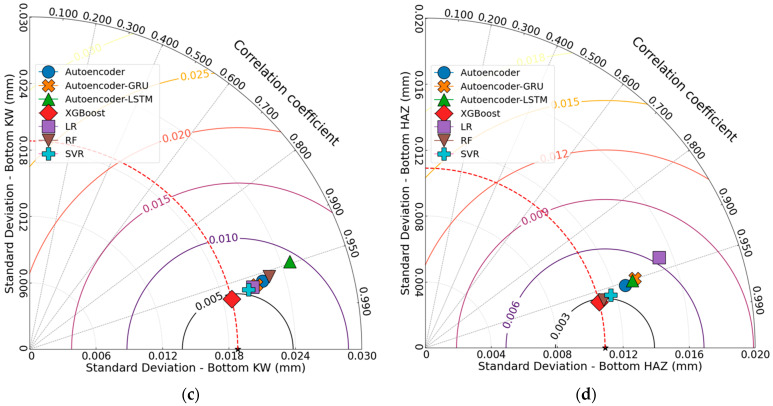
Taylor diagrams: (**a**) Ra, (**b**) Top KW, (**c**) Bottom KW, (**d**) Bottom HAZ.

Figure 19 shows the Taylor diagram of the responses. The comparative analysis shown in the figures provides a multidimensional representation of the prediction accuracy of seven ML models used for the responses of FFF-manufactured ASA parts cut by the CO_2_ laser. When examining the Taylor diagram for Ra, the XGBoost model outperformed the others, exhibiting both a high correlation coefficient and a low standard deviation (r = 0.9804, σ = 0.1408). The RF (r = 0.9778, σ = 0.1495) and Autoencoder (r = 0.9715, σ = 0.1693) models also delivered similarly consistent results. In contrast, the Autoencoder–GRU model was identified as the least successful due to its low correlation coefficient and high standard deviation (r = 0.8995, σ = 0.3120). The remaining models demonstrated moderate accuracy and consistency. For Top KW prediction, XGBoost again delivered the best performance (r = 0.9889, σ = 0.0105). This result is consistent with the findings of the study conducted by Basar [51]. The RF, SVR, and LR models also performed well, clustering close to XGBoost. Although Autoencoder and Autoencoder–GRU showed slightly lower performance, their results were still competitive. On the other hand, the Autoencoder–LSTM model (r = 0.9513, σ = 0.0223) was the least successful, with a lower correlation coefficient and higher standard deviation. Regarding Bottom KW, the best performance was achieved by the XGBoost model (r = 0.9706, σ = 0.0188), followed closely by SVR (r = 0.9649, σ = 0.0205) and LR (r = 0.9633, σ = 0.0209). While Autoencoder–GRU and Autoencoder produced competitive results, they were slightly less successful. Autoencoder–LSTM had the lowest correlation coefficient and the highest standard deviation, indicating the poorest prediction performance (r = 0.9476, σ = 0.0248). In the prediction of Bottom HAZ, XGBoost (r = 0.9670, σ = 0.0109) and RF (r = 0.9654, σ = 0.0112) were the most successful models. Notably, the LR model (r = 0.9327, σ = 0.0152) displayed a lower correlation coefficient and higher standard deviation. Overall, XGBoost was the most successful model across all responses. However, the least successful models varied depending on the response: Autoencoder–GRU for Ra, Autoencoder–LSTM for both Top KW and Bottom KW, and LR for Bottom HAZ.

Table 4 shows a comprehensive comparison of various ML models in predicting the key quality outputs. Among all models tested, the XGBoost algorithm consistently achieved superior performance across all output variables, demonstrating the lowest error rates (MSE, MAE, RMSE) and the highest goodness-of-fit indicators (R^2^ and Pearson’s correlation). Specifically, for Ra prediction, XGBoost attained an MSE of 0.019833 and an R^2^ of 0.961096, outperforming even DL-based models such as Autoencoder–GRU and Autoencoder–LSTM. For Top KW, the XGBoost model again yielded superior results with the lowest RMSE (0.010510) and the highest Pearson’s correlation (0.989), indicating excellent agreement between predicted and actual values. In terms of Bottom KW, XGBoost produced the most accurate predictions, with a remarkably low MAE of 0.015369 and an R^2^ of 0.942. Similarly, for Bottom HAZ, XGBoost showed the strongest prediction capability with the highest accuracy (MAE = 0.009128, R^2^ = 0.935), suggesting its robustness in modeling even the most thermally sensitive output. While DL architectures such as Autoencoder–LSTM and Autoencoder–GRU delivered competitive results—particularly in modeling temporal patterns and capturing nonlinear dependencies—their performance was slightly behind that of XGBoost in most cases. Based on the performance metrics, the least successful models were identified as follows: for Ra, the Autoencoder–GRU model (MSE = 0.097324, MAE = 0.253061, RMSE = 0.311969, R^2^ = 0.809117, Pearson’s correlation = 0.902966); for Top KW, the Autoencoder–LSTM model (MSE = 0.000498, MAE = 0.018920, RMSE = 0.022320, R^2^ = 0.905, Pearson’s correlation = 0.952); for Bottom KW, the Autoencoder–LSTM model (MSE = 0.000616, MAE = 0.020636, RMSE = 0.024820, R^2^ = 0.898, Pearson’s correlation = 0.948); and for Bottom HAZ, the LR model (MSE = 0.000230, MAE = 0.012738, RMSE = 0.015160, R^2^ = 0.87, Pearson’s correlation = 0.933). Overall, the results clearly indicate that ensemble-based approaches such as XGBoost are highly effective and reliable for multi-output prediction in advanced manufacturing processes such as CO_2_ laser cutting. These findings also emphasize the importance of model selection in predictive analytics for process optimization, where hybrid and non-linear models offer significant advantages over linear counterparts.

## 5. Conclusions

This research fits within the scope of studies on CO_2_ laser cutting of Fused Filament Fabricated (FFF) ASA thermoplastic parts, mainly from the perspective of multi-parameter optimization and ML-based predictive modeling of the key performance indicators. The laser cutting was performed with plate thicknesses of 2, 2.5, 3, 3.5, and 4 mm; laser powers of 90, 95, and 100 W; and cutting speeds of 3, 6, and 9 mm/s. Measurements of surface roughness (Ra), top kerf width (Top KW), bottom kerf width (Bottom KW), and bottom heat-affected zone (Bottom HAZ) were conducted for analysis. This multi-output setting provides a more integral view of how the CO_2_ laser parameters affect the quality and dimensional accuracy of FFF-printed ASA components.

The results revealed that each quality metric was primarily influenced by a different process parameter. Plate thickness emerged as the most critical factor affecting surface roughness, explaining nearly 89% of the observed variance according to ANOVA results. The best Ra value (2.998 µm) was achieved at 3.5 mm thickness, 90 W power, and 9 mm/s cutting speed. Conversely, Bottom KW was most affected by cutting speed, while laser power had the highest contribution to the Bottom HAZ formation. Top KW was influenced by a combination of all three parameters, with plate thickness having the dominant role. The results are consistent with previous studies on laser cutting of thermoplastics, but they provide a more detailed and comprehensive understanding of ASA components, particularly due to the wider range of thickness levels and the extensive performance metrics evaluated in this research. A microscopic analysis and a surface response plot illustrate more parameter interaction. Increased cutting speed decreases Ra, KW, and HAZ, mainly due to less heat accumulation and interaction time between the laser and the material. While high laser power generally induces large heat-affected zones and kerf widths, different interactions of laser power with cutting speed and plate thickness give rise to nonlinear effects. For example, at a thickness of 4 mm, increasing laser power reduces Ra, perhaps indicating that complex thermal–material dynamics are at play for ASA laser processing.

In the ML component of the study, seven models were evaluated for their ability to predict the four output variables. These included DL-based models (Autoencoder, AE-GRU, AE-LSTM), ensemble methods (Random Forest, XGBoost), and traditional models (Support Vector Regression and Linear Regression). Model performance was assessed through a robust multi-evaluation framework (MEMs) involving scatter analysis, residual plots, Taylor diagrams, and statistical error metrics. The results demonstrated that ensemble models, particularly XGBoost, consistently outperformed others across all outputs, delivering the lowest MSE, MAE, and RMSE, and the highest R^2^ and Pearson’s coefficients. XGBoost achieved an R^2^ of 0.961 for Ra, 0.978 for Top KW, 0.942 for Bottom KW, and 0.935 for Bottom HAZ. On the other hand, the unsuccessful models for each response were identified as follows: Autoencoder–GRU for Ra, Autoencoder–LSTM for both Top KW and Bottom KW, and LR for Bottom HAZ.

The innovation of this study stems from combining experimental laser cutting with ML-based prediction of multiple quality outputs in ASA processing. Unlike earlier works that primarily focused on single response parameters and limited material variations, this research combines a broad experimental dataset with advanced modeling techniques to provide a unified optimization and prediction framework. The proposed approach not only enhances the understanding of ASA’s laser machine behavior but also offers a replicable methodology for other thermoplastics processes via FFF. In brief, this research makes a valuable addition to the area of advanced manufacturing by bringing both empirical knowledge and computational tools to the forefront in the optimization of CO_2_ laser cutting processes in polymeric additive manufacturing. The findings will help practitioners select process parameters for an acceptable surface finish, dimensional accuracy, and thermal damage. Nevertheless, the prediction capability of ML models developed in this work can be extended for real-time control and adaptive laser processing in smart manufacturing systems. Future research may investigate other hybrid DL architectures and include other physical parameters, such as beam focal length or assist gas flow rate, to better their performance and generalizability.

## Figures and Tables

**Table 1:** Control factors and levels.

Factors	Level				
	1	2	3	4	5
Plate thickness, t (mm)	2	2.5	3	3.5	4
Cutting speed, v (mm/s)	3	6	9	-	-
Power, p (W)	90	95	100	-	-

**Table 2:** ANOVA results for responses.

Source	DF	Seq SS	Adj SS	Adj MS	F-Value	*p*-Value	Contribution (%)
Ra (µm)							
t	4	16.5422	16.5422	4.13555	136.11	*p* < 0.001	89.00
p	2	0.4278	0.4278	0.21389	7.04	0.003	2.30
v	2	0.5222	0.5222	0.26112	8.59	0.001	2.81
E	36	1.0938	1.0938	0.03038			5.89
T	44	18.5860					100
R^2^ = 94.11%, R^2^ (adj) = 92.81%, R^2^ (pred) = 90.80%							
Top KW (mm)							
t	4	0.030483	0.030483	0.007621	178.88	*p* < 0.001	49.26
p	2	0.013806	0.013806	0.006903	162.04	*p* < 0.001	22.31
v	2	0.016056	0.016056	0.008028	188.44	*p* < 0.001	25.95
E	36	0.001534	0.001534	0.000043			2.48
T	44	0.061879					100
R^2^ = 97.52%, R^2^ (adj) = 96.97%, R^2^ (pred) = 96.13%							
Bottom KW (mm)							
t	4	0.078303	0.078303	0.019576	98.52	*p* < 0.001	37.88
p	2	0.012193	0.012193	0.006096	30.68	*p* < 0.001	5.90
v	2	0.109063	0.109063	0.054531	274.45	*p* < 0.001	52.76
E	36	0.007153	0.007153	0.000199			3.46
T	44	0.206711					100
R^2^ = 96.54%, R^2^ (adj) = 95.77%, R^2^ (pred) = 94.59%							
Bottom HAZ (mm)							
t	4	0.010013	0.010013	0.002503	61.75	*p* < 0.001	27.30
p	2	0.016412	0.016412	0.008206	202.44	*p* < 0.001	44.75
v	2	0.008789	0.008789	0.004395	108.41	*p* < 0.001	23.97
E	36	0.001459	0.001459	0.000041			3.98
T	44	0.036673					100
R^2^ = 96.02%, R^2^ (adj) = 95.14%, R^2^ (pred) = 93.78%							

**Table 3:** Summary of model parameters and architectures.

Model	Structure/Parameters	Optimization
Autoencoder	Input(3)→Dense(16, ReLU)→Dense(1),	$\mathrm{Adam}\;(l_{r}$ = 0.001)
Autoencoder–GRU	epochs = 100, batch_size = 8	$\mathrm{Adam}\;(l_{r}$ = 0.001)
Autoencoder–LSTM	Input(1, 3)→GRU(16, ReLU)→RepeatVector→GRU(3, ReLU)→TimeDistributed(Dense(1)),	$\mathrm{Adam}\;(l_{r}$ = 0.001)
XGBoost	epochs = 100, batch_size = 8	$\mathrm{Gradient}\;\mathrm{Boosting}\;(l_{r}$ = 0.01)
LR	Input(1, 3)→LSTM(16, ReLU)→RepeatVector→LSTM(3, ReLU)→TimeDistributed(Dense(1)),	OLS (Ordinary Least Squares)
RF	epochs = 100, batch_size = 8	Bootstrap Aggregating
SVR	n_estimators = 100, learning_rate = 0.1, random_state = 42	Sequential Minimal Optimization

**Table 4:** Evaluation results of ML models.

Feature	Model	MSE	MAE	RMSE	R^2^	Pearson’s Correlation
Ra (µm)	Autoencoder	0.028647	0.132964	0.169258	0.943827	0.972846
	Autoencoder-GRU	0.097324	0.253061	0.311969	0.809117	0.902966
	Autoencoder-LSTM	0.029635	0.134807	0.172147	0.941849	0.971967
	LR	0.054040	0.187999	0.232465	0.893952	0.947262
	RF	0.022351	0.119657	0.149503	0.956137	0.978986
	SVR	0.037026	0.153715	0.192421	0.927396	0.964120
	XGBoost	0.019833	0.110816	0.140832	0.961096	0.981599
Top KW (mm)	Autoencoder	0.000189	0.011670	0.013760	0.964000	0.982000
	Autoencoder-GRU	0.000215	0.012470	0.014680	0.959000	0.980000
	Autoencoder-LSTM	0.000498	0.018920	0.022320	0.905000	0.952000
	LR	0.000177	0.011310	0.013300	0.966000	0.983000
	RF	0.000158	0.010690	0.012560	0.969000	0.985000
	SVR	0.000163	0.010860	0.012780	0.968000	0.984000
	XGBoost	0.000110	0.008980	0.010510	0.978000	0.989000
Bottom KW (mm)	Autoencoder	0.000478	0.018175	0.021870	0.921000	0.960000
	Autoencoder-GRU	0.000453	0.017671	0.021280	0.925000	0.962000
	Autoencoder-LSTM	0.000616	0.020636	0.024820	0.898000	0.948000
	LR	0.000438	0.017143	0.020930	0.928000	0.963000
	RF	0.000511	0.018547	0.022600	0.916000	0.957000
	SVR	0.000420	0.016975	0.020490	0.931000	0.965000
	XGBoost	0.000352	0.015369	0.018760	0.942000	0.971000
Bottom HAZ (mm)	Autoencoder	0.000162	0.010567	0.012735	0.911000	0.955000
	Autoencoder-GRU	0.000180	0.011173	0.013415	0.901000	0.950000
	Autoencoder-LSTM	0.000175	0.011006	0.013230	0.904000	0.951000
	LR	0.000230	0.012738	0.015160	0.870000	0.933000
	RF	0.000125	0.009355	0.011180	0.932000	0.966000
	SVR	0.000137	0.009796	0.011700	0.925000	0.962000
	XGBoost	0.000119	0.009128	0.010910	0.935000	0.967000

## Data Availability

Data is contained within this article.
